# Multiple domain insertions and losses in the evolution of the Rab prenylation complex

**DOI:** 10.1186/1471-2148-7-140

**Published:** 2007-08-17

**Authors:** Rita Rasteiro, Jose B Pereira-Leal

**Affiliations:** 1 Instituto Gulbenkian de Ciência, Apartado 14, P-2781-901 Oeiras Portugal

## Abstract

**Background:**

Rab proteins are regulators of vesicular trafficking, requiring a lipid modification for proper function, prenylation of C-terminal cysteines. This is catalysed by a complex of a catalytic heterodimer (Rab Geranylgeranyl Transferase – RabGGTase) and an accessory protein (Rab Escort Protein. REP). Components of this complex display domain insertions relative to paralogous proteins. The function of these inserted domains is unclear.

**Results:**

We profiled the domain architecture of the components of the Rab prenylation complex in evolution. We identified the orthologues of the components of the Rab prenylation machinery in 43 organisms, representing the crown eukaryotic groups. We characterize in detail the domain structure of all these components and the phylogenetic relationships between the individual domains.

**Conclusion:**

We found different domain insertions in different taxa, in α-subunits of RGGTase and REP. Our results suggest that there were multiple insertions, expansions and contractions in the evolution of this prenylation complex.

## Background

Protein prenyl transferases are a family of protein complexes that catalyze the lipid modification of proteins with isoprenoid groups. There are covalently attached to cysteine residues near or at the C-termini of intracellular proteins via tioether linkages (reviewed in [1,2]). The family includes Protein Farnesyl Transferase (FTase), Geranylgeranyl Transferase I (GGTase I) and RabGeranylgeranyl Transferase (RabGTTase). FTase and GGTase I modify C-terminal cystein residues in the context of a CAAX motif with a 15 or 20 carbon isoprenoid, respectively, and are thus termed CAAX prenyltransferases (A stands for aliphatic residue, X for any residue). Substrates of the FTase include Ras family small GTPases, nuclear lamins, centromeric proteins among others, whereas substrates of the latter include the Rho family of small GTPases and heterotrimeric G protein γ subunits (reviewed in reference [3]). RabGGTase in contrast is specific to the Rab family of small GTPases, and catalyses the modification of these with two or sometimes one 20 carbon isoprenoid. It also differs from the CAXX prenyltransferases by the absolute requirement for an accessory protein, termed Rab Escort Protein (REP) for proper catalysis (reviewed in [4]). Protein prenylation affects proteins involved in a multitude of cellular processes, is involved in a variety of human diseases and therepaeutic approaches and this is extensively reviewed, for example in references [3-6].

**Figure 1:**
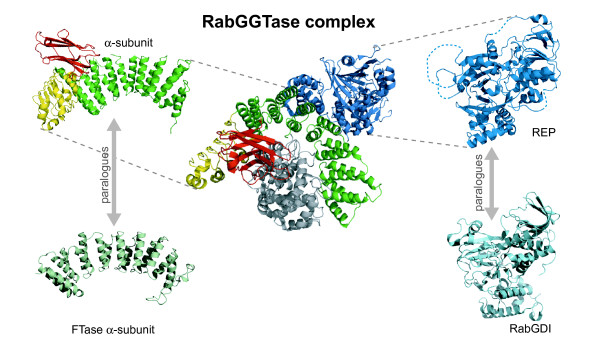
Cartoon representing the three dimensional structure of Rat RabGGTase complexed with REP-1 (1LTX) [29]. The α subunit is shown in green with a yellow and red parts, representing a Leucine Rich Repeat and a C2-like domain, respectively. REP1 is shown in blue with dotted blue lines representing disordered regions. The β subunit is shown in gray. The α-subunit and REP are shown in isolation on the left and right side respectively, next to cartoons representing the three dimensional structure of the paralogous α-subunit of FTase and of RabGDI (PDB codes 1FT1 [11] and 1LV0 [72], respectively).

Protein prenyl transferases are heterodimeric complexes of a α and a β chain, and in the case of RGGTase a third subunit, the REP protein(s). The genes coding for these enzymes have been cloned in a variety of species and shown to be essential for life [7-9]. FTase and GGTaseI share the α-subunit, but have distinct β-subunits, whereas the α- and β-subunits of RabGGTase are coded by distinct genes. The three-dimensional structure of the three enzymes has been solved, and revealed that the three enzymes are structurally homologous (see Figure 1). The α-subunit is a right-handed, crescent shaped, super-helix composed by 15 α-helices wrapped around the α-α barrel of the β-subunit [10,11]. RabGGTase is a similar αβ heterodimer but the rat protein displays the insertion of two additional domains in the α-subunit relative to the other prenyltransferases, and to the yeast protein, a Ig-like domain and a Leucine Rich Repeat domain [12]. In contrast with the CAXX prenyltransferases that recognize the C-terminal motif of the substrate proteins via the β, catalytic subunit, RabGGTase recognizes the substrate by interactions of the α-subunit with an extra protein, REP. The rat REP, like the α-subunit, displays an insertion relative to the yeast orthologue and the paralogous RabGDI [13,14] (Figure 1). Protein prenyltransferases are believed to have evolved from an ancestral heterodimer, which by gene duplication gave rise to the current constellation of subunits [15].

The role of gene duplication in the evolution of protein complexes has been subject of recent interest [16-19]. Individual gene duplications can contribute to specialization of protein complexes, and can also accumulate to the point where two or more fully independent protein complexes exist [16,19]. Less well understood is the role of domain insertions and deletions in the context of protein complexes. Protein domains are the basic evolutionary units in protein evolution [20,21]. Proteins can gain, rearrange and loose domains in the course of evolution. Domain gains result mostly from fusions of independent genes that contain one or more domains and it is perhaps the most frequent mechanism of protein evolution after gene duplication [22]. It can also be a consequence of duplication followed by in-frame fusion, resulting in tandem duplications. Less frequently, domain insertions may happen, situations where one or more domains are inserted into another domain, usually in loop regions [23,24]. Domain losses are less well understood, but recent evidence suggests that they are most abundant at C-termini and due to the premature stop codons [25]. Rearrangements or circular permutations can be achieved by complete duplication with in-frame fusion followed by partial deletion of domains at the termini, the most frequent mechanism [26], but also by independent fusion [26] and also by a mechanism termed "cut and paste" where a gene is fragmented, for example by the action of endonucleases, and then reassembles the resulting fragments in a different order [27].

Here we characterize the domain architectures of the components of the Rab prenylation complex, *i.e. *the heterodimer αβ of RGGTase and the accessory protein REP. We find that orthologous proteins display diverse domain architectures, suggestive of multiple independent events involving gains and losses of domains.

## Results and discussion

### α subunit – tetratricopeptide repeats

The rat α-subunit of RGGTase is very similar to the corresponding α-subunit of FTase, containing 15 α-helices arranged in a crescent-shaped, double layered right-handed superhelix, enveloping the β-subunit [11,12]. Its structural architecture, together with statistically significant sequence similarity with the tetratricopeptide repeat (TPR) motif indicates that it belongs of the TPR superfamily [15]. An individual TPR is a pair of anti-parallel α-helices, with consensus residues mediating the packing of these helices [15]. In the rat RGGTase α-subunit these 7 TPR are helices 2 to 15. The crystal structure of the Rat RGGTase revealed two additional domains, a C-terminal Leucine Rich Repeat domain (LRR), and an Ig-like domain inserted between helices 11 and 12, *i.e. *between TPR 5 and 6. This is the exact same loop where in the FTase α-subunit there is a short 3_10 _helix between α-helices 11 and 12 [11].

We investigated the complete and partial genome sequences of 43 organisms, covering the crown eukaryotic groups discicristates, excavates, alveolata, heterokonts, plants, amoebozoa and the opisthokonts (metazoa, fungi and choanoflagelates) [28]. In all the organisms with a complete genome sequence available, we detect a putative RGGTase α-subunit (Figure S1). Thus we can conclude that the last eukaryotic common ancestor already possessed this subunit. FTase α-subunits were also found in the majority of the organisms studied (Figure S1), suggesting that the duplication that gave rise to the two distinct α-subunits preceded the split of the eukaryotic crown groups.

Detailed phylogenetic reconstruction of the evolutionary relationships between α-subunits based solely on the TPRs shows that the α-subunits of each enzyme are monophyletic (Figure S1). It is then most likely that there was one and only one gene duplication event that created the two paralogous subunits, and that this happened at the base of the eukaryotic tree. Furthermore, the fact that the secondary structure composed of 15 repeating helices with TPRs is conserved suggests that the ancestral of prenyltransferases already had this configuration.

We find that the secondary structure of this subunit varies in evolution, displaying multiple domain insertions in different organisms, which we characterize below.

### α subunit – C2-like domain

Rat RGGTase a-subunit displays an inserted globular domain between helices 11 and 12 [12]. The function of this domain is unclear, but it is clearly not involved in contacts with REP nor the Rab substrates [29]. This globular domain is a β sandwich composed of eight strands in two sheets. It is a domain broadly related to the Ig fold, and in the two papers describing the structure of the complex it was termed an Ig-like domain. This is consistent with the CATH hierarchy, a fully automated classification of protein structures [30]. Here we will consider instead the SCOP classification of evolutionary relationships between proteins structures, as it complements automated classification with manual curation [21,31]. SCOP 1.71 classifies this domain as an independent superfamily, included in the C2 domain-like fold. From now on we will refer to this domain as the C2 domain-like.

The full SCOP hierarchy for this domain is shown in additional file 1 (Figure S2). This fold includes superfamilies such as the C2 domain. This is a domain that is found in multiple eukaryotic proteins and is involved in signaling, vesicular transport, modification of lipids, among other functions [32]. C2 domains usually regulate their respective protein function by establishing Ca^2+^-dependent and Ca^2+^-independent phospholipids complexes. One class of C2 domains can bind Ca^2+ ^without binding phospholipids [33]. Recently, the C2 domain of PKCd was shown to mediate protein-protein interactions by binding directly to phosphotyrosine peptides in a sequence-specific manner [34]. It is unclear if the RabGGTase C2-lke domain displays any of these functions. Since the role of Rab isoprenylation is to allow hydrophilic Rab proteins to associated with cellular membranes, it is plausible to think that such modification should occur in proximity to those membranes. If this C2 domain-like is a phospholipid-binding domain, then it could play a role in bringing the prenylation reaction next to membranes. Ultra-structural studies could be used to test this hypothesis.

**Figure 2:**
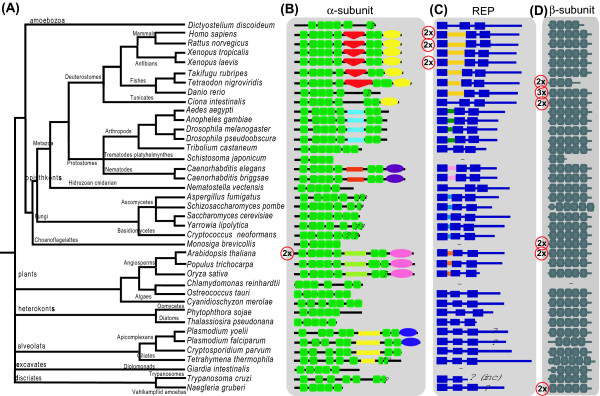
**(A) **taxonomic tree of the organisms analyzed in this study, adapted from the NCBI's taxonomy database [73] and from reference [28] **(B) **N- to C-terminal domain organization of the α-subunit of RGGTase. Boxes with the same shape represent the same domain, whereas different shapes represent distinct domains. Green boxes represent tetratricopeptide repeats (TPRs). In between the 5^th ^and 6^th ^TPR some proteins display an inserted domain. In deuterostomes this is a C2-like domain (inverted orange triangle), but in other organisms the inserted sequences are not recognized as any known domain. They are represented by colored lines. Inserts of the same color are similar to each other. Ellipses represent LRR domains – distinct colors represent sequence similarity. The red circle with 2× on the left indicates that this protein is duplicated in that organism. **(C) **N- to C-terminal domain organization of REP. The blue boxes represent the Sequence Conserved Regions (SCRs) between SCR1 and SCR2 there is an inserted domain of unknown structure. This domain is only conserved within taxa: all the inserted sequences represented in yellow are similar to each other but distinct from all other inserted sequences represented in other colors. **(D) **N- to C-terminal domain organization of the β-subunit The gray boxes represent the prenyltransferase and squalene oxidase repeat.

It was previously observed that this domain was not present in the yeast orthologue of α-subunit RGGTase, but that it would be present in worm also [15]. We analyzed in detail the sequences between helices 11 and 12 of RGGTase, where the Rat C2-like domain is found [12], and also where the FTase a-subunit displays an inserted 3_10 _helix [11]. We found that only a restricted number of branches on the eukaryotic tree display insertions in this region (Figure 2). These include metazoa, plants and alveolata. All other branches of the tree have a predicted secondary structure similar to that of Bet2 in *S. cerevisae*, which does no display any insertion between the two TPRs.

Next we investigated whether the insert we found on the three distinct branches of the tree of life are similar, and hence likely to represent one single insertion event in the ancestor of all RGGTases that was subsequently lost in other branches. Or instead whether they are distinct domains, resulting from independent insertion or expansion events. Our results favor the second hypothesis (Figure 2). We find that the inserted domains are similar within taxonomical groups, but different beyond recognition across taxa. By difference beyond recognition we mean BLAST sequence searches [35], Pfam [36] and Superfamily [37,38] domain assignments and secondary structure predictions [39,40]. Thus, in Metazoa, all Deuterostomes have a clearly defined C2-like domain, but the insects and the nematodes have a distinct insertion conserved solely in their taxonomical group. In the plants, angiosperms have a conserved domain in the same region of the α-subunit, but different beyond recognition from any other insert of the orthologous α-subunits. The same is true for alveolata. The size of the insertion is also conserved with taxonomic group but not across groups. For example, whereas deuterostomes have an insert of near 125 amino residues long, plants have a larger one, nearing 200 residues (Figure S3). Our results are thus compatible at least with up to five distinct insertion and/or expansion events in the same position of the α-subunit of RGGTase.

One exciting possibility is that all these insertions and or expansions represent the same function accomplished by different sequences, as this would expand our repertoire of sequence-function relationships.

Multiple sequence insertions/expansions in the same site suggest that this site is capable of accommodating structural variations more easily than others. Thus there seems to be a structural constraint in place. The fact that the paralogous α-subunit of FTase contains an inserted region in between these helices, in the form of a 3_10 _helix adds support to this hypothesis. However, if the function of these distinct insertions and/or expansions is the same, then this could be the only place where this function is possible, and thus the recurrent use of the same site reflect functional rather than structural constraint. In the absence of information regarding the role played by these insertions/expansions it is not possible to resolve this question.

### α subunit – LRR domain

At the C-terminus of rat RGGTase α-subunit there is a Leucine Rich Repeat domain (Figure 1). This domain in not present in FTase (Figure 1). LRRs belong to the SCOP fold of the same name, which groups proteins forming a right-handed β-α superhelix [21,31]. It is formed by three superfamilies, the RNI-like, Outer arm dynein light chain and the L domain-like which includes the RGGTase LRR domain. LRRs are involved in a variety of biological processes, both in eukaryotes and prokaryotes. Their common role is the establishment of complexes with other proteins [41,42].

**Figure 3:**
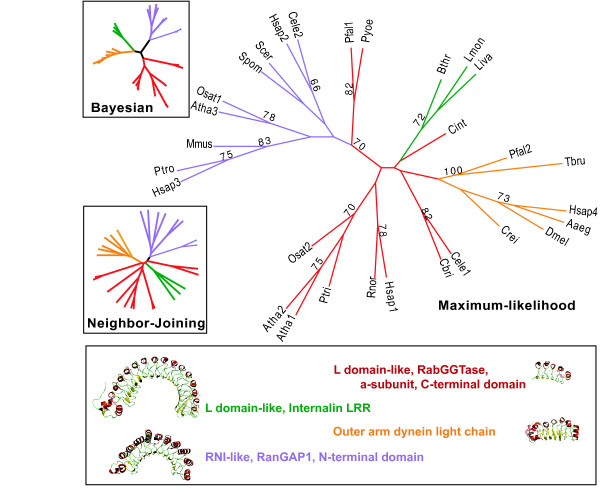
Maximum Likelihood reconstruction of the evolutionary relationships between the LRR domains found in RGGTase a-subunit (red branches) and reference sequences representing other superfamilies of LRR domains – L-domain-like, internalin LRR (106v) – green; RNI-like, 28-residue LRR, Ribonucelase inhibitor (1A4Y) – blue; RNI-like, RanGAP1, N-terminal domain(1K5D) – purple; outer arm dynein light chain(1M9L) – orange; L-domain-like, RabGGTase α-subunit, C-terminal domain (1LTX) – red. Inset boxes show the topologies of Bayesian and Neighbor Joining trees of the same sequences, showing contradicting topologies. Species codes are Aaeg – Aedes aegypti; Atha – Arabidopsis thaliana; Bthr-Bacillus thrugiensis; Cbri – Caenorhabditis briggsae; Cele – Caenorhabditis elegans; Cint – Ciona intestinalis; Crei – Chlamydomonas reinhardtii; Dmel – Drosophila melanogaster; Hsap – Homo sapiens; Liva – Listeria ivanovii, Lmon – Listeria monocytogenes; Mmus – Mus musculus; Osat – Oryza sativa; Pfal-Plasmodium falciparum; Ptri – Populus trichocarpa; Ptro – Pan troglodytes; Pyoe-Plamodium yoelii; Rnor – Rattus norvegicus; Scer – Saccharomyces cerevisae;Spom – Schizosaccharomyces pombe; Tbru – Trypanosoma brucei

We now investigate whether this LRR domain is a feature of all RGGTase α-subunits, which would suggest that it was present in the ancestral eukaryote, or if it is a recent acquisition restricted to a specific taxon or set of taxa. It is clear from the results shown in Figure 2 that the LRR domain is not universal, as we can only detect it in some animals, in angiosperms and in alveolata. This phylogenetic profile is consistent with two evolutionary scenarios – independent domain fusions or a single domain fusion at the base of the eukaryotic tree followed by a specific domain loss in multiple branches of the eukaryotic tree. Both scenarios seem equally unlikely, so we investigated this further using phylogenetic reconstruction based on the LRR domain sequences only. Our hypotheses is that there is enough phylogenetic signal in these sequences to solve this puzzle. In Figure 3 we show three phylogenies of the LRRs, including sequences from other superfamilies as reference, reconstructed by three distinct methods. Plant and vertebrate LRR are consistently monophyletic, suggesting a common origin. In contrast, the LRR of *Ciona*, of nematodes and of alveolata segregate with different reference sequences. This varies according to the method used to reconstruct phylogeny. It is thus impossible at this stage to resolve the question regarding the common or independent origin of the LRR sequences in the α-subunit. However, the recent observation that convergent evolution of domain architectures is very rare, with an estimated frequency of 0.4% to 4% [43], is more supportive of the first scenario.

### β-subunit domain architecture

The β-subunit of rat RGGTase is a α-α barrel composed of 12 α helices. It is very similar to the α-α barrel in the β-subunit of FTase [12]. The β-subunits of prenyltransferases are more conserved than the α subunits, but identification and classification of RGGTase β-subunit was simple using a combination of BLAST searches of sequences databases followed by phylogenetic analysis (Figure S4).

We discussed above that the α-subunit display multiple sequence insertions in different species. Below we will show that in some species REP also display inserted sequences. The β-subunit in contrast appears to have an invariable domain architecture throughout evolution. We investigated its structural relatives in order to gain insight whether this is due to structural constraints. In order to do so, we investigated the SCOP hierarchical classification of protein structures. Prenyl transferase's β-subunits belong to the "α-α toroid" fold (SCOP: 48207), Terpenoid cyclases/Protein prenyltransferases superfamily (SCOP: 48239). This fold consists of multihelical proteins displaying up to seven alpha-hairpins arranged in a closed circular array. Therpene synthases are classified into the same superfamily. These proteins are characterized by two α-α domains in the same peptide chain; the first is an α6-α6 barrel of two concentric rings, whereas the second is a barrel with 10 α helices, one 3_10 _helix and at least two β-strands inserted in between helical elements [44]. It appears then that this superfamily can have varying number of helical elements as well as accommodate extra structural elements. It is then plausible that the invariable nature of the domain architecture of β-subunits is due to functional rather than structural constraints.

In animals and yeast it is clear that RGGTase is composed of independent α and β-subunits. In contrast GGTase I and FTase have distinct β-subunits, but share a α-subunit. Thus the GGTase I and FTase are related by a single gene duplication. In contrast, RGGTase is separated from the other prenyltrasnsferases by two gene duplications. Although the number of duplication steps that separates the different enzymes is clear, the order of duplication is not. In other words, we don't know which subunits emerged first and which resulted from these by duplication. We find that all organisms with a complete genome sequence that we investigated display a β-subunit of RGGTase (Figure 2). Considering we also always find a RGGTase α-subunit in the same organisms, it follows that the ancestor of all eukaryotes already had a distinct and fully separated RGGTase.

### REP

**Figure 4:**
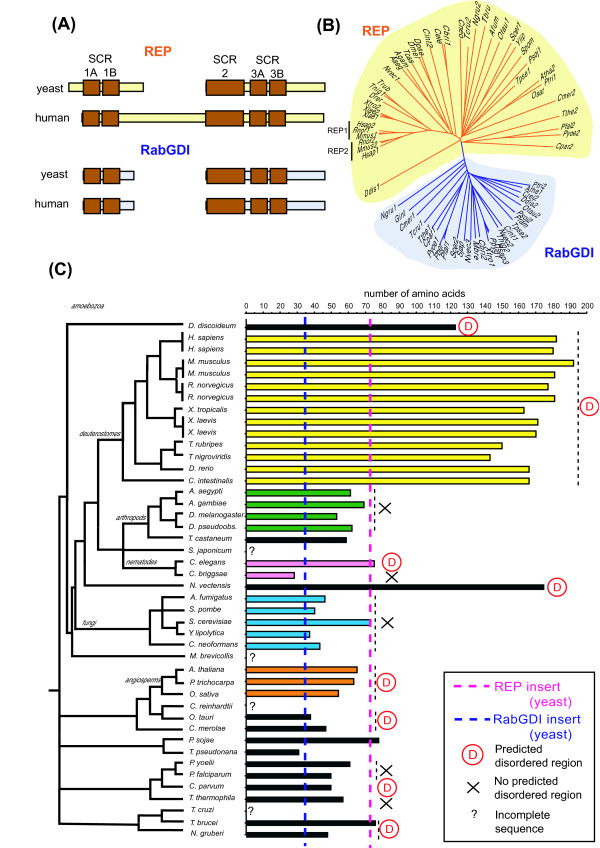
**(A) **scheme illustrating the location of the sequence conserved regions (SCRs) in RabGDI and REP sequences, as well as the variable size of the region between SCR1A and SCR2. The broken yellow and blue boxes indicate the "gap" in the alignment of these sequences with mammalian REP. Adapted from [13] **(B) **Neighbor-Joining tree of selected sequences of REP and RabGDI, illustrating that classification of these sequences into one of the two subfamilies is clear. The same tree with bootstrap vaues is provided as supplementary Figure S6. Species codes are Aaeg – Aedes aegypti; Afum – Aspergillus fumigatus; Agam – Anopheles gambiae; Atha – Arabidopsis thaliana; Cbri – Caenorhabditis briggsae; Cele-Caenorhabditis elegans; Cint – Ciona intestinalis; Cmer – Cyanidioschyzon merolae; Cneo – Cryptococcus neoformans; Cpar – Cryptosporodium parvum; Crei-Chlamydomonas reinhardtii; Ddis – Dictyostelium discoideum; Dmel – Drosophila melanogaster; Dpse – Drosophila pseudoobscura; Drer – Danio rerio; Gint-Giardis intestinalis; Hsap – Homo sapiens; Mbre – Monosiga brevicollis; Mmus-Mus musculus; Ngru – Naegleria gruberi; Nvec – Nematostella vectensis; Osat-Oryza sativa; Otau – Ostreococcus tauri; Pber – Plasmodium berghei; Pfal – Plasmodium falciparum; Ppyg-Pongo pygmaeus; Pram – Phytophthora ramorum; Psoj – Phytophthora sojae; Ptri-Populus trichocarpa; Pyoe – Plamodium yoelii; Rnor – Rattus norvegicus; Scer-Saccharomyces cerevisae; Sjap – Schistosoma japonicum; Spom-Schizosaccharomyces pombe; Tbru – Trypanosoma brucei; Tcas – Tribolium castaneum; Tcru – Trypanosoma cruzi; Tpse-Thalassiosira pseudonana; Tnig – Tetraodon nigroviridis; Trub – Takifugu rubripes; Tthe – Tetrahymena thermophila; Xlae – Xenopus laevis; Xtro – Xenopus tropicalis; Ylip – Yarrowia lipolytica **(C) **Plot of the size of the region between SCR1A and SCR2 in the different species, measured in number of amino acids. The bars are coded according to the colors used in Figure 2 and same color denotes identifiable sequence similarity. The blue and pink dotted perpendicular lines are for reference and indicate the number of amino acids between SCR1B and SCR2 for RabGDI and MRS6, respectively. A red D indicates that the insert contains a predicted disordered region [51], whereas a cross means that such regions are not predicted in the insert.

Rab escort proteins (REP) belong to the same protein family as Rab GDP dissociation inhibitors (RabGDI). They are both classified in the SCOP hierarchy [31] as FAD/NAD(P)-binding domain fold and superfamily, which suggests a common ancestry. Their structure comprises two domains: domain I include the Rab binding platform, whereas domain II in REP mediates binding to the alpha-subunit of RGGTase [29,45]. REP and RabGDI share conserved regions, termed SCRs (sequence conserved regions), which are highlighted in Figure 4 as brown boxes. Multiple sequence alignments of REPs and RabGDI reveal that mammalian REPs display an insertion between domain I and domain II, absent in RabGDI, which maps roughly to a sequence region delimited by the conserved regions SCR1B and SCR2 [13,45]. This insert appears to be larger in vertebrate sequences than in S. cerevisae [13]. The function of this insert is unclear, particularly at the light of the recent structure of REP1 in complex with RGGT, which shows that it is not involved in contacts with the RGGTase subunits nor with Rab substrates [29].

We identified REPs in all species studied here by searching GeneBank and the other genomic databases (see methods) with known REP sequences. We used phylogenetic reconstruction to classify the obtained sequences into the REP and RabGDI subfamilies (Figure 4B). We identified at least one distinct REP sequence in all species for which a complete genome sequence was available. Mammals have two paralogous REPS (REP-1 or Choroideremia and REP-2 or CHM-like). *X. laevis *also displays two paralogous REPs, but our phylogenetic analysis suggests that this is an independent and species-specific duplication (Figure 4b). It is interesting to note that in *A. thaliana*, the duplication of both enzyme subunits was not accompanied by REP duplication.

The most striking observation is that few branches of the tree of life are characterized by the presence of a larger insert region between SCR1B and SCR2 in REPs. Their size indicates that few independent taxa display inserts that are larger than those of yeast MRS6 (yeast REP) and RabGDIs (Figure 4C). At the sequence level there is no real conservation – the inserts are similar within taxa but very different across taxa. Systematic database searches using solely the insert regions from several specie can only find closely related REP sequences. For example, a BLAST search using the insert region of rat or human REP1 will find only deuterostomes REP inserts, but not plant inserts, and *vice versa*. They are also not similar to any other protein other than REPs, which means that we cannot use this approach to define hypothetical functions for this region.

In the crystal structure of rat REP1, this sequence insert corresponds to a region in the crystal with no clear electron density [29]. Such regions are typically labeled natively unfolded [46,47]. Natively unfolded proteins are involved in a variety of cellular functions, namely transcriptional and translational regulation, signaling and regulation of the self-assembly of large multi-subunit complexes such as the ribosome and the bacterial flagellum [47,48]. They are also expected to be involved in a variety of human diseases [49]. Although they can perform their function in the unfolded state, the majority of unfolded regions undergo a process termed induced-folding, in which upon binding to their physiological partners they undergo a transition to a structured form [50]. We tested whether the different inserts were also natively unfolded, using the predictive algorithm Globplot2 [51]. We observed that angiosperm insert sequences are predicted to include a disordered, region, so are all vertebrate sequences and nematodes. In contrast, insects never show a predicted disordered region within the insert region (Figure 4C and S5). Thus, the presence of disordered regions is not restricted to the larger inserts of vertebrates, nor does it seem to correlate with insert size.

### Duplications

In most species studied, we find a single copy of the α- and the β-subunit. In contrast we found two RGGTase α- and β-subunits in *A. thaliana*. We have previously observed that duplication of whole protein complexes is frequent [16]. We argued for the prevalence of stepwise duplications leading to complete duplication of subunits of complexes, like what is observed with the adaptin tetrameric complexes [52]. The duplication of the two subunits of RGGTase in *A. thaliana *however appears to have occurred simultaneously, as a result of a whole genome duplication (WGD) estimated to have happened around 38 million years ago [53]. The paralogous pairs localize to distinct chromosomes, to duplicated segments that were mapped to that WGD (α-subunit: At4g2424490 (chr.4) and At5g41820 (chr.5); β-subunit: At3g12070 (chr.3) and At5g12210 (chr.5)).

The functional relevance of maintaining these two copies of RGGTase is unclear. It is clear from all the phylogenetic trees that the subunits are very closely related (e.g. Figure S1), suggesting some selective pressure. *A. thaliana *may require high levels of this enzyme and concerted gene duplication is one way of boosting the levels of a given gene product [54]. Alternatively, the large number and diversity of Rab GTPases in *A. thaliana *[55,56] may require specific regulation of Rab prenylation. This could be achieved by having multiple copies of the enzyme subunits under differential regulation or displaying distinct substrate specificities.

Animals also display an expansion of the Rab family. They don't have multiple copies of the enzyme subunits; they have instead two paralogous REPs that appear to form complexes with distinct substrates and have distinct specificities to the RGGTase [57]. This lends some support to the second hypothesis. It would be thus interesting to investigate whether there is specificity in the pairing of the *A. thaliana *subunits, and whether the different versions of the enzyme have distinct substrate specificities. In contrast, some animals display two or more copies of the β-subunit for a single α-subunit (Figure 2). Since substrate-binding specificity is not defined by the β-subunit in RabGGTase, it seems plausible that expression levels are at the root of these duplications. Amoebozoa like *D. discoideum*, which represents another example of independent expansions of the Rab family, have in excess of 50 Rab proteins (unpublished observations). *D. discoideum *only has one copy of each component (Figure 2), indicating that large Rab families are compatible with a single copy of each RabGGTase component.

The duplication of components of RabGGTase appear thus to be restricted to specific branches of the tree of life (Figure 2). Our analysis also indicates that it is also restricted to RabGGTase. We did not observe duplications of FTase nor GGTase I subunits. This is despite the fact that their substrates show expansions akin to those of the Rab family. For example, the Ras and Rho families expanded from 3 and 6 members respectively in *S. cerevisiae*, to 22 and 34, respectively, in *H. sapiens *[58]. It is possible that dosage balance may place a barrier to duplications of subunits [18,59].

## Conclusion

In summary, we identified the orthologues of the components of the Rab prenylation complex in 43 species – the αβ heterodimer RabGGTase, and the accessory proteins REP. We characterized their domain architectures and found that it varies considerably in evolution. The α-subunit can have distinct inserts in two positions, between the tetratricopeptide repeats 5 and 6 and at the C-terminal. These domain insertions are specific to RabGGTase, as the paralogous α-subunit of Farnesyl and Geranylgeranyl (type I) Transferases displays conserved domain architectures. The β subunit has a conserved domain architecture but the REP proteins also have variable inserts between SCR1B and SCR2.

We found the full constellation of protein prenyltransferases in the organisms we analyzed as found in model organisms [1,2]. This suggests that the Last Common Eukaryotic Ancestor (L.E.C.A.) had the same constellation of enzymes and subunits. The three enzymes are related by gene duplication events, which suggests that there was an ancestral heterodimeric protein prenyl transferase at the base of the eukaryotic tree. The presence of structural homologues of α and β-subunits in all the branches of the tree of life indicates that the eukaryotic innovation required solely novel interactions, rather than novel folds. The precise order of duplication events is however unclear. FTase and GGTase I are separated by a single gene duplication event, whereas RGGTase is separated by two duplication events from either of the other enzymes. The simpler architecture of FTase and GGTase I, and their independence from further components for proper activity suggests that they preceded RGGTase. However, substrates of all three enzymes are ubiquitous in the eukaryotic tree, which further emphasizes that the L.E.C.A. is likely to have already the three enzymes.

In conclusion, the components of the Rab prenylation complex display varied domain architectures in evolution, which are more consistent with multiple independent events in the first insertion of the α-subunit, multiple losses in the second insert, and independent expansions in REP. There is no known function for the variable domains. There is no obvious correlation between Rab family size, and the presence or size of any of the RGGTase inserts, which suggests that these insertions are independent of Rab family expansion. Furthermore, the inserted domains do not parallel each other within or across subunits. This  indicates that it is unlikely that they are involved in direct physical contacts, and that they are likely to have independent functions. The few inserted sequences that are similar to known sequences (C2-like and LRR) suggest that the role of these domains is regulatory, possibly involving lipid and protein binding. It is tempting to speculate that they are involved in taxon-specific regulatory interactions not yet described. One future avenue of research will be the identification of putative binding partners, for example by bioinformatic analysis, searching for genes with similar phylogenetic profiles as those of the inserted domains.

Finally, this case study suggests that domain gains and losses may be an important force driving the evolution and diversification of protein complexes. A future avenue of research is the quantification of this contribution.

## Methods

The analyses were performed on sequenced eukaryotic genomes that were downloaded from NCBI and other eukaryotic genomes databases. A full listing of the organisms investigated is provided in additional file 1. It aims for maximum coverage of eukaryotic diversity within the species with a complete genome sequence. Sequence searches combined BLAST [35] searches using known RGGTase and REP sequences as query, as well as protein families defined in the Pfam [36] and superfamily [37,38] databases. Multiple sequence alignments were performed with ClustalW 1.83 [60], pairwise alignments used the Smith-Waterman algorithm [61] (Water in EMBOSS [62]), in both cases with the a Blosum 62 matrix [63] and default GAP and extension penalties. Sequence manipulation was done with Jalview 2.1.1 [64]. Domain assignments were done using Superfamily [65] and Pfam [66]. Secondary structure predictions was performed using Jpred [39,40]. Phylogenetic reconstruction was done using the Neighbor-Joining clustering algorithm as implemented in ClustalW 1.83 [60], using 1000 bootstraps, as well as the cladistic methods Maximum Likelihood in the Phylip 3.61 package (ProML) [67] (Jones-Taylor-Thorthon (JTT) matrix; 100 boostraps) and Bayesian method implemented in MrBayes v. 3.1.2 [68,69] using Blosum62 as a fixed rate aa model, ran until average standard deviation of split frequencies was lower than 0.01. Trees were drawn using in FigTree v. 1.0 [70].

All sequences used in this study, as well as alignment and tree files can be found in the supplementary website [71].

## Authors' contributions

JBPL conceived the study and wrote the manuscript. RR and JBPL performed the analysis.

## Supplementary Material

Additional file 1 Supplementary material. supplementary figures and tables Click here for file
