# Supplementary material for: Multiple domain insertions and losses in the evolution of the Rab prenylation complex
Source: BMC Evol Biol. 2007 Aug 17;7:140. doi: 10.1186/1471-2148-7-140 (PMC1994686; doi:10.1186/1471-2148-7-140)
Supplement: Additional file 1 — Supplementary material. supplementary figures and tables [file 1471-2148-7-140-S1.pdf]

Supplementary material for

Multiple domain insertions and losses in the evolution of the Rab prenylation complex

Rita Rasteiro & José B. Pereira-Leal

Organisms studied

Table S1 – Organisms studied and accession numbers of the sequences analysed.

Species	RabGGTase, Alpha	RabGGTase, Beta	REP
A. aegypti	EAT48358	EAT47544	EAT44271
A. fumigatus	XP_751767	XP_749064	XP_747864
A. gambiae	XP_318802	XP_321847	XP_311529
A. thaliana	CAB79359 and AT5G41820	NP_974770 and At3g12070	AAN03631
C. briggsae	CBP23154	CAE70140	CAE69183
C. elegans	NP_500367	NP_741214	NP_001022928
C. intestinalis	ENSCING00000018133	e_gw1.2814.1.1, estExt_genewise1.C_chr_09q0165	fgenesh3_pg.C_chr_02q000991
C. merolae	CMT460C	CMT217C	CMI215C
C. neoformans	XP_568534	XP_570274	XP_569500
C. parvum	XP_628673	XP_627541	EAK90443
C. reinhardtii	Chlre2_kg.scaffold_16000010	?	?
D. discoideum	XP_637415	XP_635670	XP_641266
D. melanogaster	NP_649512	NP_524894	AAD16891
D. pseudoobscura	EAL28557	XP_001356153	EAL25321
D. rerio	XP_692527	CAM15110, CAM15111 and CAM15112	NP_982286
G. intestinalis	XP_779849	XP_769329	?
H. sapiens	NP_004572	AAA91473	CAA45979, CAA55011
M. brevicollis	gw1.3.380.1	e_gw1.3.368.1, estExt_Genewise1.C_40326	?
M. musculus	Q9JHK4	AAI32474	Q9QXG2, NP_067325
N. gruberi	e_gw1.12.63.1	estExt_fgeneshHS_	

		pg.C_790030, fgeneshNG_pg.scaffold_98000009	
N. vectensis	fgenes1_pg.scaffold_190000005	gw.217.15.1	e_gw.2.90.1
O. sativa	NP_001058357	NP_001062217	NP_001042697
O. tauri	e_gw1.14.00.219.1	fgenesh1_pm.C_Chr_11.0001000002	CAL57457
P. berghei	XP_674021	?	?
P. falciparum	PF14_0403	XP_966015	AAN35570
P. pygmaeus	Q5NVK5	?	?
P. ramurum	fgenes1_pg.C_scaffold_7000066	?	?
P. sojae	estExt_fgenesh1.pg.C_140055	C_scaffold_27000021	estExt_fgenesh1_pg.C_2400002
P. trichocarpa	eugene3.00061586	fgenes4_pm.C_LG_XII000054	gw1.256.25.1
P. yoelii	Q7RL7	C_scaffold_27000021	PY06809
R. norvegicus	1LTX:A	1LTX:B	AAA87626, XP_001058878
S. cerevisae	YJL031C	YR176C	YOR370C
S. japonicum	AAX27330	AAW26368	?
S. pombe	NP_588463	NP_593383	CAA18894
T. castaneum	XP_974717	XP_969750	XP_966637
T. cruzi	XP_816212	XP_817693	AA023058
T. nigroviridis	CAG10947	CAF90043, CAF95466	CAF97265
T. pseudonana	genewise.1.590.1	estExt_thaps_ua_kg.C_chr_50085	thaps3_ua_kg.chr_2000181
T. rubripes	e_gw2.13.478.1	e_gw2.80.52.1	e_gw2.51.140.1
T. thermophila	EAR97274	XP_001022770	XP_001016584
X. laevis	AAH77401	AAH90236	AAH78011, AAH61662
X. tropicalis	CAJ82877	ENSXETG00000002979	fgenesh1_pg.C_scaffold_384000021
Y. lipolytica	XP_503911	XP_504498	XP_505064

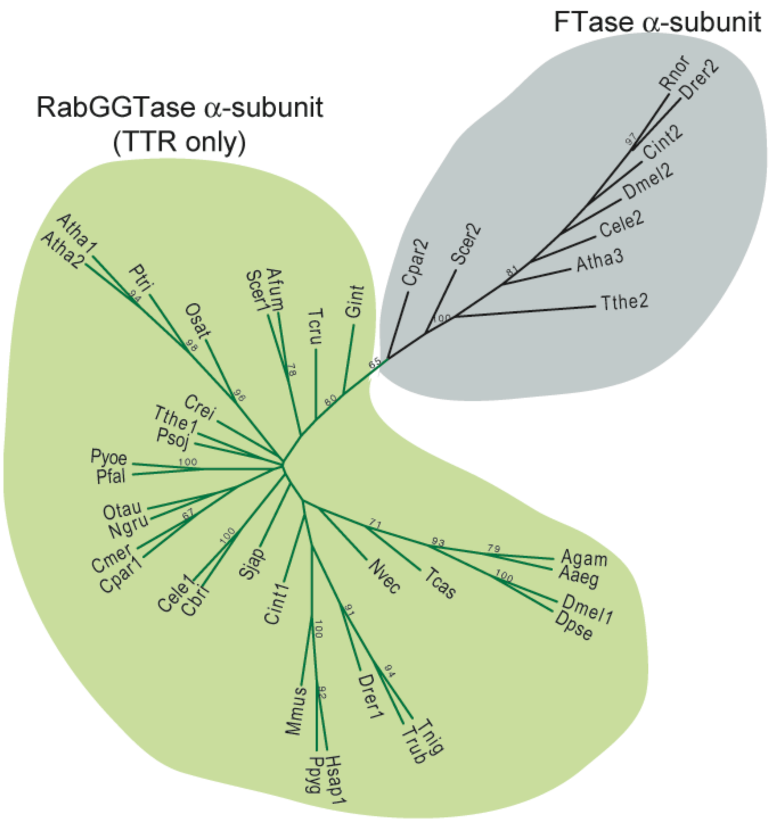

Figure S1 – Maximum Likelihood tree of the TPR regions of the α-subunits of RGGTase, and selected sequences of FTase α-subunits. Numbers next to the branches represent bootstrap support as percentage, and only when the support his higher than 65% is it shown. Species codes are Aaeg- Aedes aegypti; Afum- Aspergillus fumigatus; Agam- Anopheles gambiae;Atha- Arabidopsis thaliana; Cbri- Caenorhabditis briggsae; Cele-Caenorhabditis elegans; Cint- Ciona intestinalis; Cmer- Cyanidioschyzon merolae; Cpar- Cryptosporodium parvum; Crei- Chlamydomonas reinhardtii; Dmel- Drosophila melanogaster; Dpse- Drosophila pseudoobscura; Drer- Danio rerio; Gint- Giardis intestinalis; Hsap- Homo sapiens; Mmus- Mus musculus; Ngru- Naegleria gruberi; Nvec- Nematostella vectensis; Osat- Oryza sativa; Otau- Ostreococcus tauri; Pfal- Plasmodium falciparum; Ppyg- Pongo pygmaeus; Psoj- Phytophthora sojae; Ptri- Populus trichocarpa; Pyoe- Plamodium yoelii; Rnor- Rattus norvegicus; Scer- Saccharomyces cerevisae; Sjap- Schistosoma japonicum; Tcas- Tribolium castaneum; Tcru- Trypanosoma cruzi; Tnig-Tetraodon nigroviridis; Trub- Takifugu rubripes; Tthe- Tetrahymena thermophila

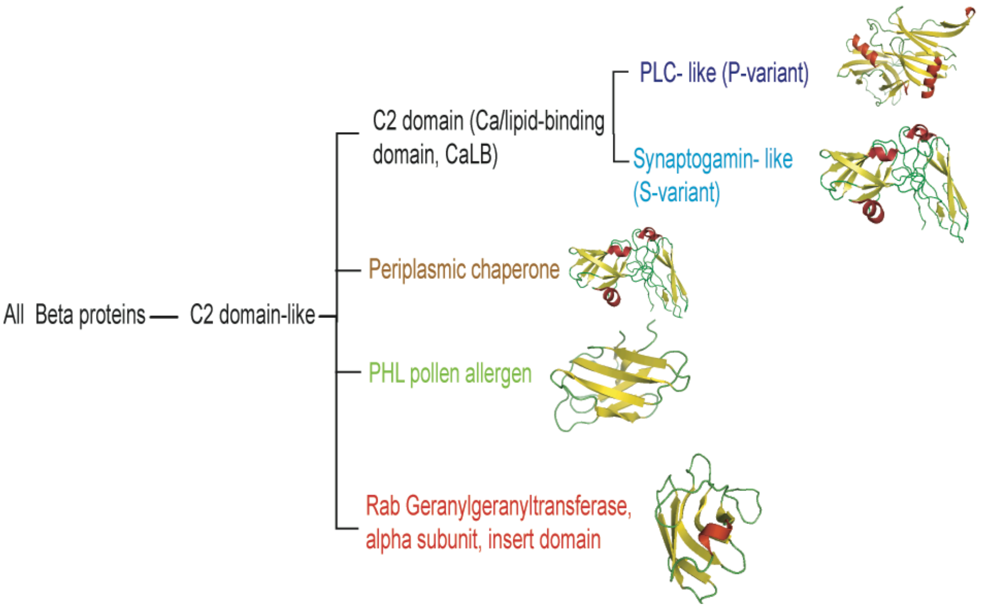

Figure S2 – SCOP hierarchy of the “C2-domain like” superfamily

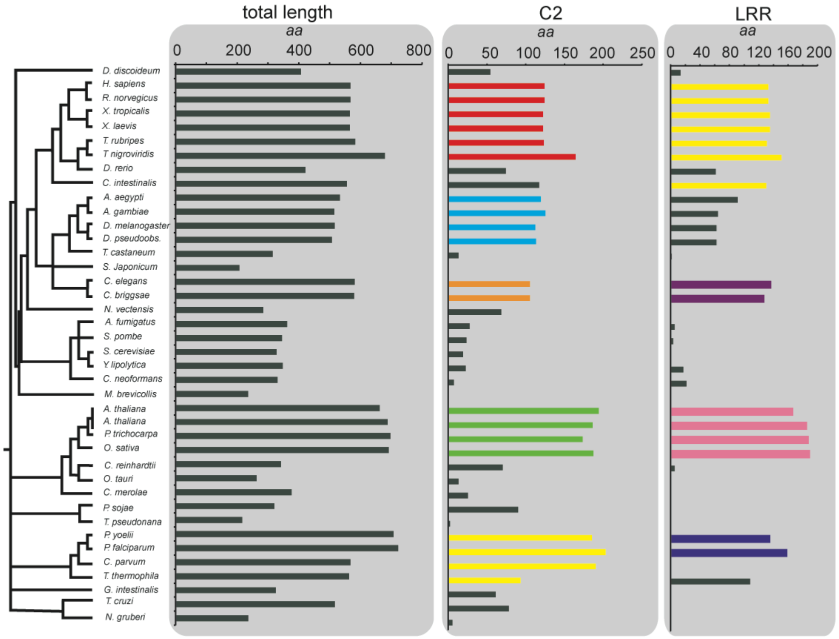

Figure S3 - Length of the α-subunit and of the regions corresponding in rat sequences to the C2-like and LRR domains. The color of the bars corresponds to the colors in Figure 2 and represents sequence similarity. The grey bars in the LRR region represent sequences not recognizable as LRR.

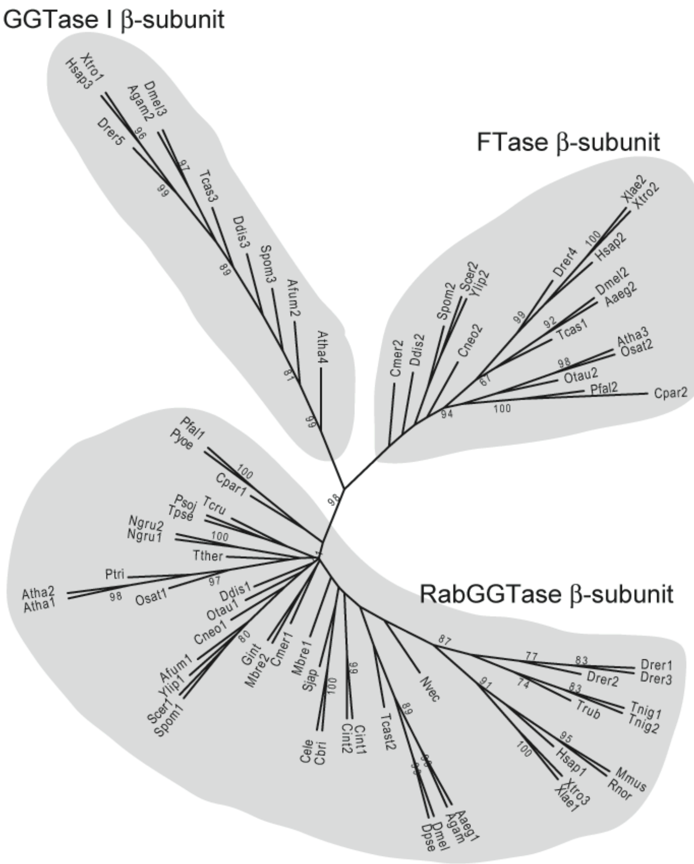

Figure S4 – Maximum Likelihood tree of β-subunit sequences. Species codes are Aaeg- Aedes aegypti; Afum- Aspergillus fumigatus; Agam- Anopheles gambiae; Atha- Arabidopsis thaliana; Cbri- Caenorhabditis briggsae; Cele- Caenorhabditis elegans; Cint- Ciona intestinalis; Cmer- Cyanidioschyzon merolae; Cneo- Cryptococcus neoformans; Cpar- Cryptosporodium parvum; Ddis- Dictyostelium discoideum; Dmel- Drosophila melanogaster; Dpse- Drosophila pseudoobscura; Drer- Danio rerio; Gint- Giardis intestinalis; Hsap- Homo sapiens; Mbre- Monosiga brevicollis; Mmus- Mus musculus; Ngru- Naegleria gruberi; Nvec- Nematostella vectensis; Osat- Oryza sativa; Pfal- Plasmodium falciparum; Psoj- Phytophthora sojae; Ptri- Populus trichocarpa; Pyoe- Plamodium yoelii; Rnor- Rattus norvegicus; Scer- Saccharomyces cerevisae; Sjap- Schistosoma japonicum; Spom- Schizosaccharomyces pombe; Otau- Ostreococcus tauri; Tcas- Tribolium castaneum; Tcru- Trypanosoma cruzi; Tpse- Thalassiosira pseudonana; Tnig- Tetraodon nigroviridis; Trub- Takifugu rubripes; Xlae- Xenopus laevis; Xtro- Xenopus tropicalis; Ylip- Yarrowia lipolytica

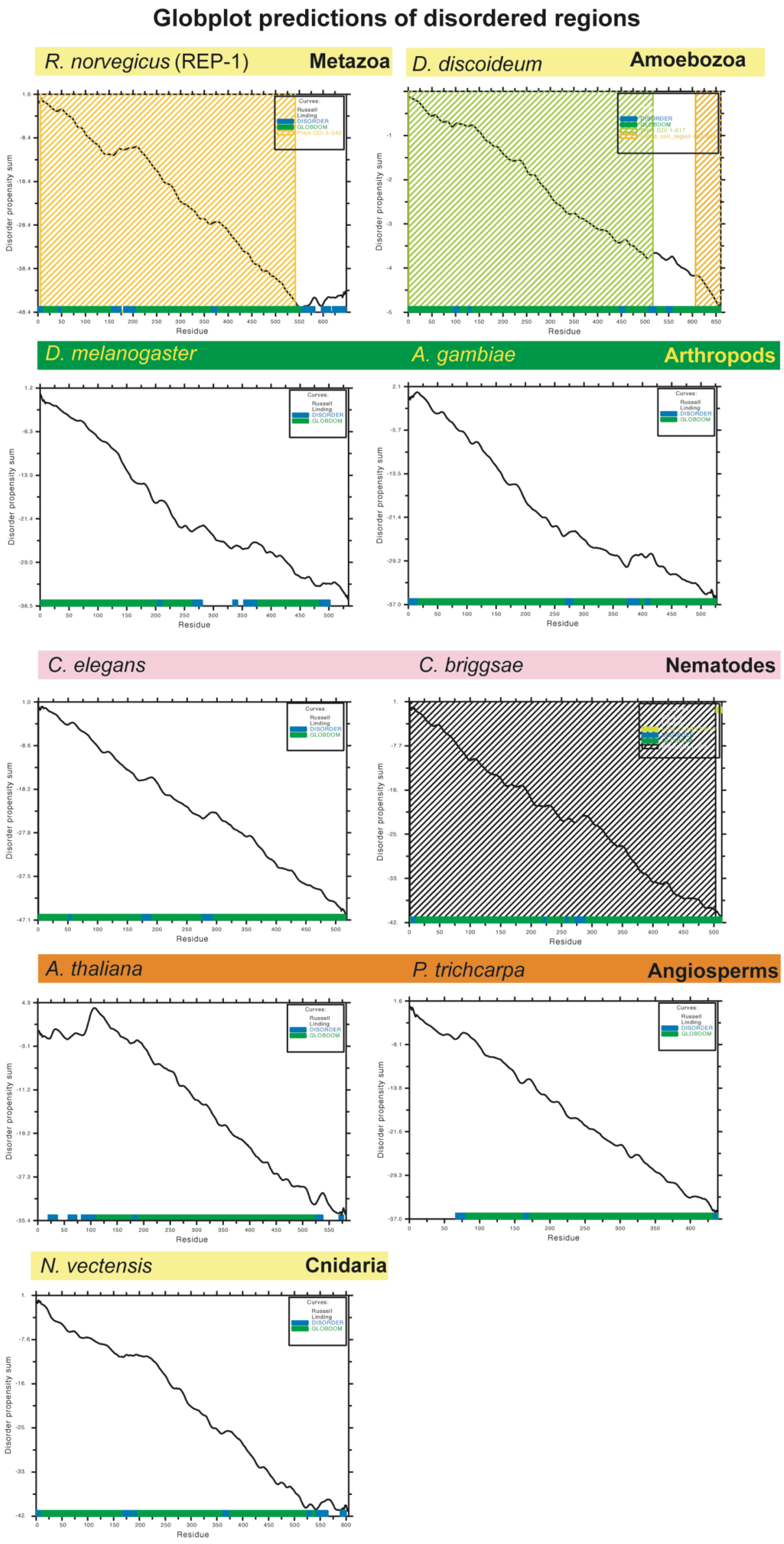

Figure S5 – Globplot results for selected REP sequences

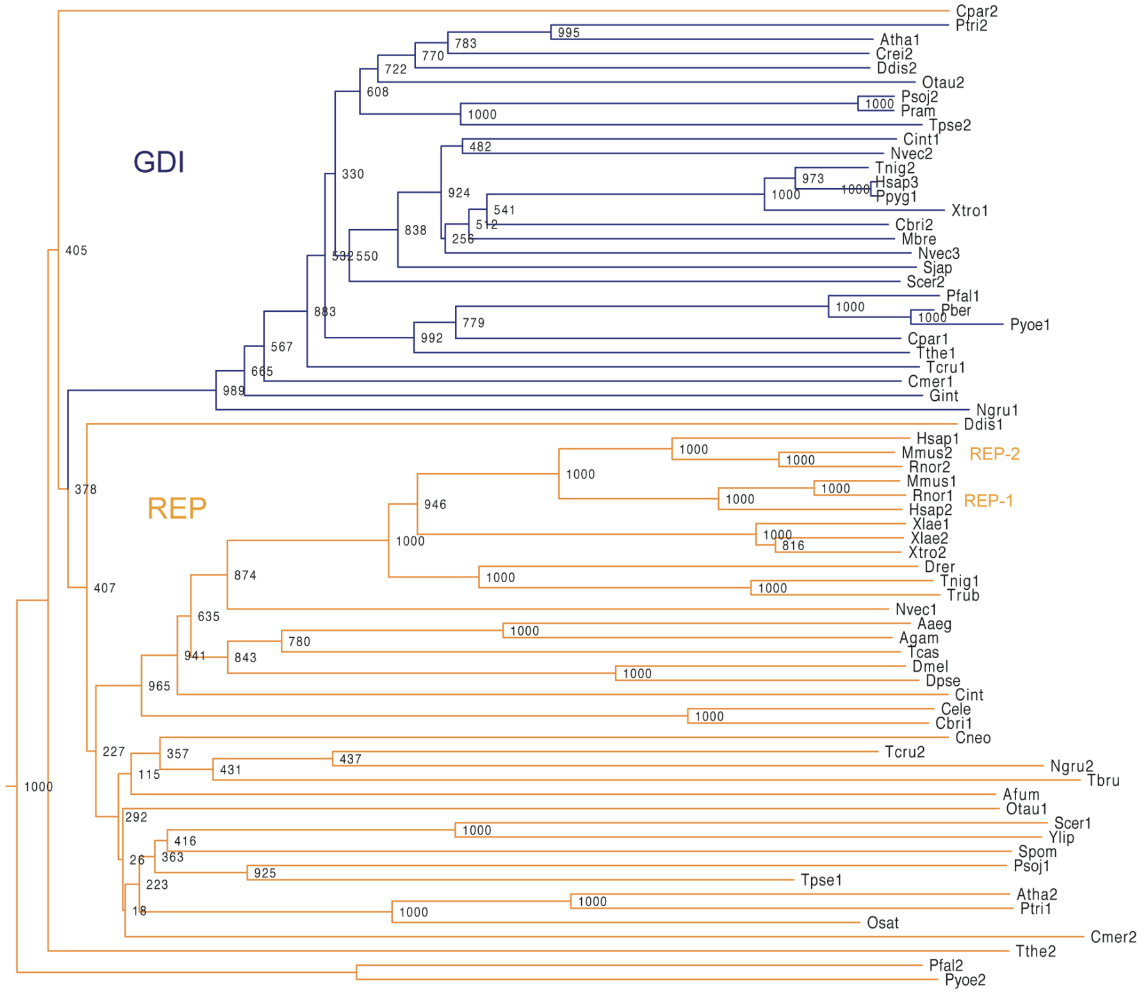

Figure S6. Neighbor-Joining tree shown in figure 4b, displaying the bootsrap values. We ran 1000 bootstraps. Species codes are the same as in Figure 4b.
